# Biofilm Formation Capacity and Presence of Virulence Determinants among *Enterococcus* Species from Milk and Raw Milk Cheeses

**DOI:** 10.3390/life13020495

**Published:** 2023-02-10

**Authors:** Joanna Gajewska, Wioleta Chajęcka-Wierzchowska, Zuzanna Byczkowska-Rostkowska, Morteza Saki

**Affiliations:** 1 Department of Industrial and Food Microbiology, Faculty of Food Science, University of Warmia and Mazury in Olsztyn, Plac Cieszyński 1, 10-726 Olsztyn, Poland; 2 Department of Microbiology, Faculty of Medicine, Ahvaz Jundishapur University of Medical Sciences, Ahvaz, Iran

**Keywords:** *Enterococcus* spp., biofilm, virulence factors, raw milk, raw-milk cheeses

## Abstract

Bacterial biofilm is one of the major hazards facing the food industry. Biofilm-forming ability is one of the most important virulence properties of enterococci. The genus *Enterococcus* includes pathogenic, spoilage, and pro-technological bacteria. The presence of enterococci in milk and dairy products is usually associated with inadequate hygiene practices. The study examined the isolates’ capacity for biofilm formation and identification of the genetic determinants of its formation among 85 *Enterococcus* strains isolated from raw milk (n = 49) and soft-ripened cheeses made from unpasteurized milk (n = 36). *E. faecalis* and *E. faecium* were the dominant species. The obtained results showed that 41.4% isolates from milk and 50.0% isolates from cheeses were able to form biofilm. All of the isolates analyzed had at least one of the studied genes. As regards the isolates from raw milk, the most prevalent gene was the *gel*E (85.6%), followed by the *asa*1 (66.7%). None of the isolates from cheeses showed the presence of *cyl*A and *spr*E. The most prevalent gene among the strains from this source was the *epb*C (94.4%), followed by the *gel*E (88.9%). In isolates from both sources, the presence of proteins from the Fsr group was noted the least frequently. Nevertheless, results showed that were no significant differences between the biofilm-producing *Enterococcus* spp. and non-biofilm-producing isolates in term of occurrences of tested virulence genes. The ability to produce a biofilm by enterococci isolated from raw milk or ready-to-eat products emphasizes the need for continuous monitoring of the mechanisms of microbial adhesion.

## 1. Introduction

Bacteria of the genus *Enterococcus* are a microorganism group widespread in the natural environment. Their common occurrence in water or soil results in the contamination of raw materials and products of both animal and plant origin. The causes of contamination of plant raw materials with these microorganisms include improper natural fertilization or the use of fecally contaminated water to irrigate crops. However, the contamination of raw materials of animal origin is caused by animal breeding and slaughtering under inappropriate conditions with disregard for the basic rules of hygiene [1,2]. Due to their high resistance to temperature, enterococci are regarded as an indicator of the hygiene of the food production process. Nevertheless, pursuant to Commission Regulation (EC) No. 2073/2005 of 15 November 2005 on microbiological criteria for foodstuffs, the determination of the presence of these microorganisms in food is not required in European Union countries [3]. Due to the high survival rate of the *Enterococcus* spp. cells, the food production process does not condition the elimination of all microbial cells from the raw material. As a result, these bacteria become part of the residual microflora of the finished products [4]. This concerns even highly processed products whose production involves many stages [5].

The biofilm-forming ability is among the most important virulence properties of the *Enterococcus* [6,7]. Biofilm is a population of cells attached to various biotic and abiotic surfaces and is encapsulated in a hydrated matrix of exopolymeric substances, proteins, polysaccharides, and nucleic acids. The biofilm structure provides an optimal environment for bacterial growth and facilitates the transfer of mobile genetic elements between different bacterial species [8]. Many proteins are involved in biofilm formation by the *Enterococcus*. The literature data indicate that the specific Enterococcal Surface protein Esp is crucial for the biofilm formation by these strains. Esp is associated with the bacterial cell wall, as it is involved in cell adhesion to the surface. *Enterococcus faecalis* strains with mutations in the *esp* gene lose their ability to produce a biofilm under in vitro conditions [9]. However, other studies suggest that the presence of *esp* is not necessary for the biofilm formation in *E. faecium* and *E. faecalis* strains [10,11]. Another protein reported to be involved in the biofilm formation is gelatinase (GelE), which can hydrolyze gelatin, collagen, and hemoglobin [6,12]. However, despite these reports, the authors demonstrated no association between biofilm formation and the presence of the *gelE* [13]. A study by Chuang-Smith and colleagues [14] showed that the presence of an aggregating substance (Agg) among the *E. faecalis* species promoted biofilm formation. In addition, the following are involved in the biofilm formation: *E. faecalis* endocarditis-associated antigen A (EfaA), adhesion of collagen of *E. faecalis* (Ace), biofilm on plastic operon (Bop), and serine protease (SprE) [15]. Additionally, in their work, Nallapareddy et al. [15] also demonstrated that the *epb*A, *ebp*B, and *ebp*C (endocarditis and biofilm-associated pili genetic) form the *ebp*ABC operon, and the *srt* (pilus-associated sortase) were genes that substantially affected the biofilm formation in *E. faecalis* strains.

An important role in biofilm formation is played by the phenomenon of *quorum sensing*, which is regulated by the *fsr* (fecal streptococci regulator) operon containing three genes: *fsr*A, *fsr*B, and *fsr*C [16]. It is worth noting that besides *quorum sensing*, bacteria of the genus *Enterococcus* spp. can communicate with each other using peptide pheromones encoded by the *cpd*, *cob*, and *ccf*.

Biofilm formation by microorganisms found in the food production chain is among the major hazards facing the food industry. The presence of biofilms on work surfaces may result in the contamination of food products with both spoilage and pathogenic microorganisms [17]. Many *Enterococcus* strains can form biofilms on food contact surfaces, which improve bacteria’s resistance to environmental stress [18]. It is worth noting that the modernity of the equipment used in the dairy industry and, consequently, the long-lasting production cycles and the associated extensive surfaces of materials in direct contact with foods, provide favorable conditions for the unhindered formation and development of a bacterial biofilm.

In view of the above, the study aimed to assess the biofilm-producing ability of the *Enterococcus* spp. strains isolated from raw milk and cheeses made from unpasteurized milk, and to identify the genetic factors responsible for biofilm formation.

## 2. Materials and Methods

### 2.1. Sample Collection and Identification of Enterococci Strains

The material for the study comprised 85 isolates identified to genus *Enterococcus*, belonging to the Department of Industrial and Food Microbiology collection. They were isolated from raw milk and raw milk cheeses using standard protocols. Strain identification was performed using a matrix-assisted laser desorption ionization time-of-flight mass spectrometry (MALDI-TOF MS) instrument (Biomerieux) according to manufacturing instructions, as described previously [19,20].

### 2.2. Congo Red Agar (CRA) Assay

The investigation of slime production by the Congo red agar assay was determined using a method described by Freeman et al. [21]. Fresh, 24 h colonies were streaked into CRA plates. The plates were incubated for 24 h at 37 °C. On the basis of colony color, the strains were classified as follows black—as slime producers, Bordeaux, or red—as non-slime-producing strains.

### 2.3. Biofilm Production by Microtiter Plate Assay (MTP)

The ability to form biofilm was tested on 96-well, flat-bottomed, sterile polystyrene plates (Promed^®^) according to Stepanović et al. [22], as described previously [19,23]. Absorbance at 570 nm wavelength was measured with spectrophotometric microplate reader Varioscan LUX (Thermo Scientific, Waltham, MA, USA). Wells containing broth only were used as negative control. Optical densities (ODs) for each test strain were determined from the arithmetic mean of 3 replicates. The value obtained was compared with the cut-off value (ODc). ODc is defined as three standard deviations above the mean OD of the negative control. Based on the results, the isolates were classified as: non-biofilm producers (OD ≤ ODc); weak biofilm producers (ODc < OD ≤ 2 × ODc); moderate biofilm producers (2 × ODc < OD ≤ 4 × ODc); and strong biofilm producers (4 × ODc < OD).

### 2.4. Detection of Biofilm-Associated Genes and Virulence Factors

The biofilm-associated genes and virulence factors were amplified by polymerase chain reaction (PCR) using published specific primers and conditions [16,24,25,26,27,28] (Table 1). Amplified products were resolved by 1.5% agarose gel electrophoresis in 1× TBE (Tris-borate-EDTA) buffer stained by 0.5μg/mL of ethidium bromide (0.5 mg/mL; Sigma-Aldrich) and visualized using the system for the documentation and analysis of fluorescently stained gels G-BOX F3 (Syngene. Cambridge, UK).

### 2.5. Statistical Analysis

All statistical analyses were performed using GraphPad Prism software version 8.0 (GRAPH PAD software Inc., San Diego, CA, USA). Results were considered statistically significant with *p* < 0.05.

## 3. Results

### 3.1. Identification of Enterococci Strains

Using the MALDI-TOF MS technique, 85 isolates from raw milk and raw milk cheeses were classified as *E. faecalis* (71; 83.5%), *E. faecium* (10; 11.8%) *E. gallinarum* (3; 3.5%), and *E. casseliflavus* (1; 1.2%) Among the strains isolated from both sources, the *E. faecalis* species was dominant (Table 2).

### 3.2. Biofilm and Slime-Forming Ability

A strong biofilm was formed by 36 (73.5%) milk isolates. None of the strains formed a weak biofilm, while one isolate (2%), belonging to the species *E. faecalis*, formed a moderate biofilm. No ability to form a biofilm was exhibited by 24.5% of raw milk isolates (Table 3). In half of the *Enterococcus* spp. isolated from raw milk cheeses (n = 18; 50.0%), no biofilm-producing ability was observed, while 17 isolates (47.2%) produced a strong biofilm. None of the cheese isolates produced a moderate biofilm (Table 3). It is noteworthy that the majority of strains belonging to the species *E. faecium* (n = 4; 80.0%) from cheeses produced a strong biofilm, while the *E. faecium* from raw milk failed to exhibit this ability. It is noteworthy that the species *E. casseliflavus* and *E. gallinarum* were able to form biofilm. Nevertheless, no statistically significant differences were found between the enterococci species and the ability to produce biofilm (*p* = 0.913202) and ability for slime production (*p* = 0.68592).

The slime-forming ability, determined by the CRA method, was detected among 21/42.9% and 20/55.6% strains isolated from raw milk and raw milk cheeses, respectively (Table 3). As for the strains *E. faecalis* and *E. galinarum*, a different tendency to produce a slime on the CRA was shown. As compared to the isolates from raw milk, this ability was exhibited by 55.2% and 100.0% of the isolates, respectively. The statistical analysis showed no correlation between the biofilm production ability of the MTP method and slime production (*p* = 0.616472).

### 3.3. Presence of Biofilm-Associated and Virulence Genes

The results obtained from molecular analysis of different biofilm-associated and virulence determinants are presented in Table 4. The most prevalent gene among all tested enterococci strains was the gelatinase-encoding *gel*E (81.7%, n = 74/85). The *esp* protein, considered by many researchers to be responsible for the biofilm formation, was found in approximately one third (32.9%, n = 28/85) of all the studied enterococcal isolates. The least prevalent proteins among all the isolates were *sprE*, *cylA*, and *fsrA*, with frequencies of 1.2% (n =1/85), 2.4% (n = 2/85), and 7.1% (n = 6/85), respectively.

Among strains from raw milk, the *gel*E was demonstrated in 85.7% of the *E. faecalis* and 80% of *E. faecium isolates*, as well as in *E. casseliflavus* and *E. gallinarum* isolates isolated from raw milk. Another highly prevalent gene found among the isolates from raw milk was the *asa*1, demonstrated in 66.7% of *E. faecalis* strains and in 50% of other species. Among strains from this source, the least frequent genes were *cyl*A, *fsr*B, and *spr*E, with frequencies of 4.1%, 6.1%, and 2.4%, respectively.

Among enterococcal strains from cheeses, our results showed that the most prevalent gene was the *epbC*, which was detected in 94.4% of all isolates. In detail, 96.6% of *E. faecalis*, 80.0% of *E. faecium*, and 100% of *E. gallinarum* isolates. The second most prevalent protein among the isolates from this source was the *gelE* (88.9%) of all isolates. Those with the lowest frequency among isolates from this source were the *fsrA* and *fsrB* operon genes responsible for the quorum sensing in bacteria of this genera. The presence of *cylA* and *sprE* was not demonstrated in any of the studied isolates from cheese samples.

It is worth noting that for this source of isolation, the statistical analysis showed an association exclusively between the presence of the *esp* and the biofilm-producing ability (*p* < 0.05).

Moreover, having considered the source of isolation (raw milk vs cheeses from unpasteurized milk) of all strains from the genus *Enterococcus* spp., an association between the source of isolation and the presence of studied genes *ebpC*, *pil*, *srt*, and *sprE* was revealed at the significance level α = 0.05. Additionally, the results showed that there are no significant differences between the biofilm-producing *Enterococcus* spp. and non-biofilm-producing isolates in term of occurrences of virulence genes (Table 5). In addition, correlation analysis for all pairs (between virulence genes and biofilm formation) among all tested enterococci has been shown in Figure S1. 

Biofilm producers were recognized as strong, intermediate, and weak biofilm-producing strains.

## 4. Discussion

Enterococci represents one of the most controversial groups of bacteria. *Enterococcus* genus is highly prevalent in foods, especially those of animal origin. Enterococci are characterized by resistance to different conditions during food production and storage, and their high adaptability [29]. The increasing enterococcal resistance to antimicrobial agents and the ability to form a biofilm crucially increases the pathogenic potential of this genus. Moreover, biofilm formation can also act as persistent sources of contamination, leading to hygiene problems in food products [30].

For many years there has been a lack of literature data on the biofilm production capacity of *Enterococcus* spp. isolated from food. Most studies on enterococci have focused exclusively on clinical strains isolated from implants, catheters, or human bodily fluids [16,31]. Given the ubiquity of enterococci in the environment and their resistance to adverse environmental conditions, they have become the predominant microflora responsible for contamination of food. However, due to the increasing resistance of these strains to antimicrobial agents, combined with their remarkable ability to acquire and transfer virulence genes, many researchers have also started to consider food as a source of virulent enterococcal strains [32,33]. Despite recent findings that enterococcal pathogenesis is a strain-dependent trait and occurs more frequently in clinical enterococci than in foodborne enterococci], it is worth emphasizing that enterococcal species do not have a status generally recognized as safe (GRAS) [34].

It is alarming that in the food production environment, where bacteria have ideal conditions for their development, biofilm formation has the potential for transferring resistance factors [35]. The lack of appropriate cleaning and disinfection procedures to be applied immediately after the technological process largely contributes to bacterial growth in the form of biofilms in which, after reaching a critical thickness, bacterial cells may detach and migrate into the environment [36,37]. These bacteria can easily migrate long distances along the production line, which results in equipment failure and food spoilage and can pose a health hazard if they reach batches of food distributed among consumers. The presence of other pathogens such as *Staphylococcus aureus* in cheeses can increase the virulence of *E. faecalis*, which is a major problem [2]. Therefore, it is important to understand the mechanism responsible for enterococcal adhesion, to prevent biofilm formation on the surfaces of the food industry equipment in the future and thus the contamination of the finished products with virulent strains.

*Enterococcus* spp. are one of the most common lactic acid bacteria in raw milk, with *E. faecium* and *E. faecalis* being predominant [30], which can come from animals, dairy environment, and humans [4]. In the current study, the dominant species in both sources of isolation was *E. faecalis* followed by *E. faecium*, which coincides with the results of other studies [33,38,39]. However, Fuka et al. [40] isolated more *E. faecalis* strains from raw milk samples, with the artisan cheese samples mostly being a source of strains of the species *E. faecium*.

In recent years, biofilm has become a worldwide public health. According to the literature data, it is worth noting that many *Enterococcus* strains can build biofilms on food contact surfaces [18]. In the current study, 73.5% isolates from raw milk and 47.2% isolates from cheeses exhibited great biofilm-forming ability. Some authors concluded that the biofilm-forming ability was more prevalent among *E. faecalis* isolates than among other species [41,42,43]. However, this is not reflected in the results obtained in the current study.

Bacterial virulence factors may be either colonization factors, like those promoting bacterial adhesion to the host cells, or an invasive factor which promotes the invasion of epithelial cells that disrupt the immune system. Several surface proteins anchored in the cell wall are involved in enterococcal pathogenicity, including the aggregating substance, the enterococcal surface protein, and collagen-binding components [44]. Proteins such as hyaluronidase can interact with lymphocyte receptors and induce autoimmune diseases. Cytolysin, on the other hand, is an exotoxin with bifunctional bacteriocin and hemolytic activity [45,46].

According to Di Rosa et al. [47], biofilm formation can be modulated by environmental conditions, gelatinase activity, and the presence of the *esp* gene. However, it is worth stressing that the joint action of several parameters (time, temperature, nutrients, genetic factors) can be more critical compared to a single factor [48,49]. However, in the light of numerous literature data, *gelE* has been recognized as a factor unrelated to biofilm formation [13,50,51,52], which was confirmed by the results of the current study. Moreover, a statistical analysis of the obtained results demonstrated, for both isolates from raw milk and cheeses, a relationship between the biofilm formation and the presence of the *esp* gene. Despite this, these results are not consistent with previous reports on the biofilm formation by the isolates lacking this gene [11,53]. The lack of biofilm-forming ability among half of the enterococci isolated from cheeses may be due to the presence of sodium chloride in a cheese (up to 4%) and its high acidity [54].

A variety of virulence factors have been described in enterococci, mostly in *E. faecalis* and *E. faecium* [55]. The enterococcal virulence factors may be classified into two groups: surface proteins and secreted metabolites that damage the host’s tissues [4,52]. Among them we can distinguish, e.g., gelatinase (*gelE*, *fsrA*, *fsrB*, *fsrC*, *sprE*), adhesins (*ace*), aggregating substance (*agg*, *asa1*), cytolysin operon (*cylA*), endocarditis and biofilm-associated pili (*ebpA*, *ebpB*, *ebpC*), and sex pheromones (*cpd*, *cob*, *ccf*) [23,56]. In the current study, the most frequently found virulence gene was the *ebpC* gene that co-participates in the formation of the pili, which was followed, in terms of prevalence, by the *gelE* gene responsible for the production of gelatinase [57]. Gomes et al. [39] demonstrated that most genetic virulence determinants (*gelE*, *esp*, *ace*, *efaA*, *cylA*) occurred with a higher frequency among *E. faecalis* strains. Moreover, these genes were more prevalent in isolates from cheeses than those from raw milk. Nevertheless, the results of the study demonstrated no correlation between the occurrences of these genes, depending on the species of *Enterococcus* spp. In a study by Fuka et al. [40], the strains isolated from milk samples were more virulent than the strains derived from cheeses. However, in a study conducted by Pereira and colleagues [41], the most frequently found virulence genes included *ace*, *agg*, and *gelE*. Moreover, these authors demonstrated that they were much more frequently found in *E. faecalis* strains.

It is worth emphasizing that the occurrence or absence of a gene does not directly indicate that the encoded protein plays a role in the ability of enterococcal strains to form a biofilm. Each stress factor during food preservation and/or food processing could cause changes in the virulence of the strain. Among stress factors, chemical factors (organic acids, ethanol, and salt) or physical factors (high and low temperature, high pressure) may be mentioned [58]. The literature data indicate that the biofilm formed on equipment surfaces in the dairy industry adversely affects the safety of the finished product. Bacterial cells present in raw milk can attach to, and grow on, the surfaces of dairy equipment. The presence of milk residues in milking equipment and storage tanks contributes to the adhesion of cells and bacterial growth [59,60]. The results of this study confirmed that *Enterococcus* spp. can produce biofilm, which is one of the fundamental problems for the dairy industry. Of note, *Enterococcus* spp. genus does not have GRAS status. Therefore, in the food chain, the pathogenic potential of enterococci is of concern due to their ability to form biofilm and the occurrence of virulence genes, along with their potential to harbor and transfer virulence to other pathogens based on horizontal gene transfer.

## 5. Conclusions

Our results showed that *E. faecalis* was a predominant species among *Enterococcus* spp. isolated from raw milk and cheese samples. The obtained results showed that 41.4% isolates from milk and 50.0% isolates from cheeses were able to form biofilm. Moreover, the results of this study showed that the presence of pathogenic factors such as the *gel*E, *cyl*A, *agg*, *ebp*A, *ebp*B, *pil* among milk isolates, and *asa*1, *ebp*C, *srt*, *fsr*A, *fsr*B among cheeses isolates, did not seem to be necessary or sufficient for the formation of biofilm by enterococci in the analyzed conditions. Enterococci are not highly virulent. Nevertheless, the results of our study suggest that it is important to control and monitor *Enterococcus* isolates that harbor adhesion genes to improve food and food production safety.

## Figures and Tables

**Table 1:** Oligonucleotides used in polymerase chain reaction.

Gene	Primers Sequence	PCR Annealing Temperature (°C)	Amplicon Size (bp)	References
*agg*	CACGTAATTCTTGCCCACCA	55	520	[24]
	CAAGCATTATTGGCAGCGTT			
*ebpA*	CCATTTGCAGAAGCAAGAATG	54	613	[16]
	GAGTGAAAGTTCCTCCTCTAG			
*ebpB*	CATTAGCAGAGGCATCGCAA	54	504	
	CAAGTGGTGGTAAGTCATAGG			
*ebpC*	CTGCTACGAATATGGTGGTG	54	487	
	GGTGTTTGATTGTTTGCTTC			
*pil*	GAAGAAACCAAAGCACCTAC	54	620	
	CTACCTAAGAAAAGAAACGCG			
*srt*	GTATCCTTTTGTTAGCGATGC	54	612	
	TGTCCTCGAACTAATAACCGA			
*fsrA*	ATGAGTGAACAAATGGCTATTTA	49	740	[25]
	CTAAGTAAGAAATAGTGCCTTGA			
*fsrB*	GGGAGCTCTGGACAAAGTATTATCTAACCG	63	566	
	TTGGTACCCACACCATCACTGACTTTTGC			
*sprE*	TTGAGCTCCGTTCCTGCCGAAAGTCATTC	55	591	
	TTGGTACCGATTGGGGAACCAGATTGACC			
*esp*	AGATTTCATCTTTGATTCTTGG	56	510	[26]
	AATTGATTCTTTAGCATCTGG			
*gel*E	TATGACAATGCTTTTTGGGAT	56	213	[27]
	AGATGCACCCGAAATAATATA			
*asa*1	GCACGCTATTACGAACTATGA	56	375	
	TAAGAAAGAACATCACCACGA			
*cyl*A	ACTCGGGGATTGATAGGC	56	688	[28]
	GCTGCTAAAGCTGCGCTT			

**Table 2:** Identification of enterococci isolates.

	Number (%) of Isolates				
Isolation Source	*E. feacalis*	*E. feacium*	*E. gallinarum*	*E. casseliflavus*	Total
Raw milk	42 (89.4%)	5 (10.2%)	1 (2.0%)	1 (2.0%)	49 (57.6%)
Raw milk cheeses	29 (80.6%)	5 (13.9%)	2 (5.6%)	0 (0.0%)	36 (42.4%)
Total	71 (83.5%)	10 (11.8%)	3 (3.5%)	1 (1.2%)	85 (100.0%)

**Table 3:** Results of biofilm-producing ability among enterococci isolated from raw milk and cheese samples.

	Milk Samples					
	Biofilm Formation				Congo Red Agar Method	
Species	Strong	Moderate	Weak	No Biofilm	Positive	Negative
*E. faecalis* (n = 42)	33 (78.6%)	1 (2.4%)	0 (0.0%)	8 (19.0%)	19 (45.2%)	23 (54.8%)
*E. faecium* (n = 5)	1 (20%)	0 (0.0%)	0 (0.0%)	4 (80%)	1 (20%)	4 (80%)
*E. casseliflavus* (n = 1)	1 (100.0%)	0 (0.0%)	0 (0.0%)	0 (0.0%)	1 (100%)	0 (0.0%)
*E. gallinarum* (n = 1)	1 (100.0%)	0 (0.0%)	0 (0.0%)	0 (0.0%)	0 (0.0%)	1 (100%)
All enterococci (n = 49)	36 (73.5%)	1 (2.0%)	0 (0.0%)	12 (24.5%)	21 (42.9%)	28 (57.1%)
	**Cheese samples**					
*E. faecalis* (n = 29)	12 (41.4%)	0 (0.0%)	1 (3.4%)	16 (55.2%)	16 (55.2%)	13 (44.8%)
*E. faecium* (n = 5)	4 (80.0%)	0 (0.0%)	0 (0.0%)	1 (20.0%)	2 (40.0%)	3 (60.0%)
*E. gallinarum* (n = 2)	1 (50.0%)	0 (0.0%)	0 (0.0%)	1 (50.0%)	2 (100.0%)	0 (0.0%)
All enterococci (n = 36)	17 (47.2%)	0 (0.0%)	1 (2.8%)	18 (50.0%)	20 (55.6%)	16 (44.4%)

**Table 4:** The presence of biofilm-associated and virulence genes in enterococci isolated from raw milk and cheese samples.

Genes	Milk Samples					Cheese Samples				
	*E. faecalis* (n = 42)	*E. faecium* (n = 5)	*E. casseliflavus* (n = 1)	*E. gallinarum* (n = 1)	Total (n = 49)	*E. faecalis* (n = 29)	*E. faecium* (n = 5)	*E. gallinarum* (n = 2)	Total (n = 36)	Total (n = 85)
*gelE*	36 (85.7%)	4 (80.0%)	1 (100.0%)	1 (100.0%)	42 (85.7%)	25 (86.2%)	5 (100.0%)	2 (100.0%)	32 (88.9%)	74 (87.1%)
*esp*	13 (31.0%)	2 (40.0%)	0 (0.0%)	0 (0.0%)	15 (30.6%)	8 (27.6%)	4 (80.0%)	1 (50.0%)	13 (36.1%)	28 (32.9%)
*asa1*	28 (66.7%)	2 (40.0%)	1 (100.0%)	0 (0.0%)	31 (63.3%)	19 (65.5%)	4 (80.0%)	2 (100.0%)	25 (69.4%)	56 (65.9%)
*cylA*	2 (4.8%)	0 (0.0%)	0 (0.0%)	0 (0.0%)	2 (4.1%)	0 (0.0%)	0 (0.0%)	0 (0.0%)	0 (0.0%)	2 (2.4%)
*agg*	12 (28.6%)	2 (40.0%)	0 (0.0%)	0 (0.0%)	14 (28.6%)	13 (44.8%)	0 (0.0%)	1 (50.0%)	14 (38.9%)	28 (32.9%)
*ebpA*	27 (64.3%)	5 (100.0%)	1 (100.0%)	1 (100.0%)	34 (69.4%)	18 (62.1%)	5 (100.0%)	2 (100.0%)	25 (69.4%)	59 (69.4%)
*ebpB*	25 (59.5%)	3 (60.0%)	1 (100.0%)	1 (100.0%)	30 (61.2%)	21 (72.4%)	5 (100.0%)	1 (50.0%)	27 (75.0%)	57 (67.1%)
*ebpC*	16 (38.1%)	1 (20.0%)	0 (0.0%)	1 (100.0%)	18 (36.7%)	28 (96.6%)	4 (80.0%)	2 (100.0%)	34 (94.4%)	52 (61.2%)
*pil*	17 (40.5%)	1 (20.0%)	0 (0.0%)	1 (100.0%)	19 (38.8%)	26 (89.7%)	3 (60.0%)	2 (100.0%)	31 (86.1%)	50 (58.8%)
*srt*	20 (47.6%)	4 (80.0%)	0 (0.0%)	1 (100.0%)	25 (51.0%)	23 (79.3%)	4 (80.0%)	1 (50.0%)	28 (77.8%)	53 (62.4%)
*fsrA*	0 (0.0%)	0 (0.0%)	0 (0.0%)	0 (0.0%)	0 (0.0%)	5 (17.2%)	1 (20.0%)	0 (0.0%)	6 (16.7%)	6 (7.1%)
*fsrB*	1 (2.4%)	2 (40.0%)	0 (0.0%)	0 (0.0%)	3 (6.1%)	7 (24.1%)	1 (20.0%)	0 (0.0%)	8 (22.2%)	11 (12.9%)
*spr*E	1 (2.4%)	0 (0.0%)	0 (0.0%)	0 (0.0%)	1 (2.0%)	6 (20.7%)	0 (0.0%)	0 (0.0%)	6 (16.7%)	7 (8.2%)

*gel*E: gelatinase; *esp*: enterococcal surface protein, *asa*1: aggregation substance, *cyl*A: cytolysin operon, *agg*: aggregation substance; *ebp*A, *ebp*B, *epb*C: endocarditis and biofilm-associated pili; *pil*: pili; *srt*: pilus-associated sortase; *fsr*A: response regulator; *fsr*B: signaling peptide; *spr*E: serine protease.

**Table 5:** Association between the occurrences of virulence genes and biofilm formation ability of all enterococci isolates, both from milk and cheeses.

Virulence Genes	*Enterococcus* Species (n = 85)		
	Biofilm Producers (n = 55)	Non-Biofilm Producers (n = 30)	*p*-Value
	n (%)	n (%)	
*gelE*	48 (87.3)	26 (86.7)	1.000
*esp*	19 (34.5)	9 (30.0)	0.810
*asa1*	36 (65.5)	21 (70.0)	0.810
*cylA*	2 (3.6)	0 (0.0)	0.537
*agg*	16 (29.1)	12 (40.0)	0.341
*ebpA*	36 (65.5)	23 (76.7)	0.332
*ebpB*	34 (61.8)	23 (76.7)	0.228
*ebpC*	30 (54.5)	22 (73.3)	0.107
*pil*	29 (52.7)	21 (70.0)	0.167
*srt*	31 (56.4)	22 (73.3)	0.162
*fsrA*	2 (3.6)	4 (13.3)	0.179
*fsrB*	7 (12.7)	4 (13.3)	1.000

## Data Availability

Not applicable.

## Supplementary Materials

The following supporting information can be downloaded at: https://www.mdpi.com/article/10.3390/life13020495/s1, Figure S1: Correlation between all pairs of tested virulence genes, slime production and biofilm formation among all tested enterococci.
