# Supplementary material for: Biofilm Formation Capacity and Presence of Virulence Determinants among Enterococcus Species from Milk and Raw Milk Cheeses
Source: Life (Basel). 2023 Feb 10;13(2):495. doi: 10.3390/life13020495 (PMC9962698; doi:10.3390/life13020495)
Supplement: Supplementary file 1 [file life-13-00495-s001.zip › life-2158586-supplementary.pdf]

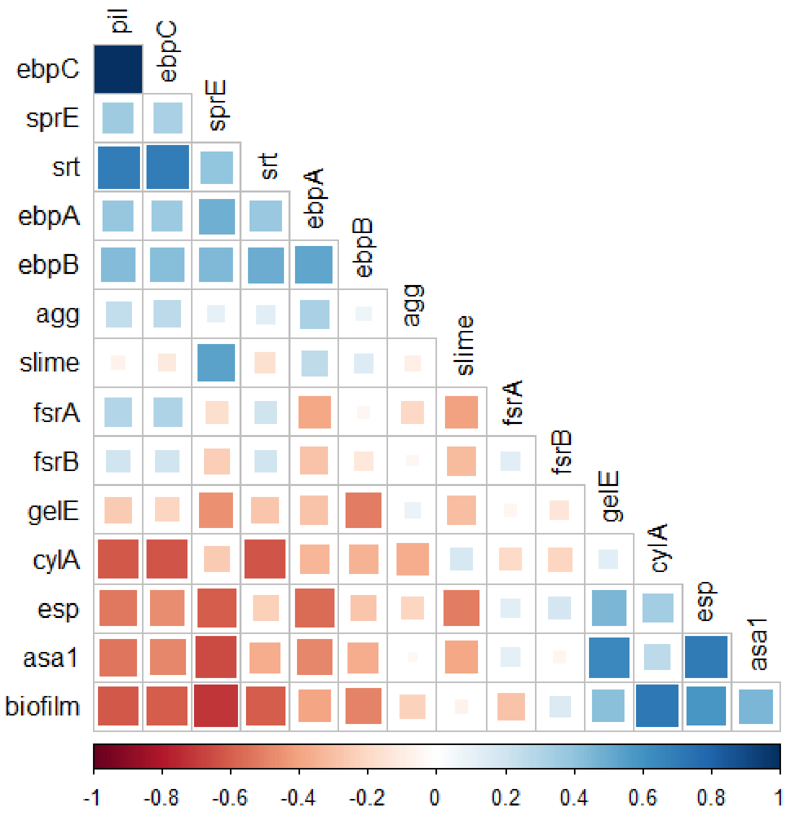

Figure S1. Correlation between all pairs of tested virulence genes, slime production and biofilm formation among all tested enterococci.
